# Molecular Characterization of *Echinococcus granulosus* Cysts in North Indian Patients: Identification of G1, G3, G5 and G6 Genotypes

**DOI:** 10.1371/journal.pntd.0002262

**Published:** 2013-06-13

**Authors:** Monika Sharma, Rakesh Sehgal, Bashir Ahmad Fomda, Anil Malhotra, Nancy Malla

**Affiliations:** 1 Department of Parasitology, Postgraduate Institute of Medical Education and Research, Chandigarh, India; 2 Department of Microbiology, Sher-i Kashmir Institute of Medical Sciences, Srinagar, Jammu and Kashmir, India; 3 Department of Surgery, Indira Gandhi Medical Collage, Shimla, Himachal Pradesh, India; University of Zurich, Switzerland

## Abstract

**Background:**

Cystic echinococcosis (CE) caused by the *Echinococcus granulosus*, is a major public health problem worldwide, including India. The different genotypes of *E. granulosus* responsible for human hydatidosis have been reported from endemic areas throughout the world. However, the genetic characterization of *E. granulosus* infecting the human population in India is lacking. The aim of study was to ascertain the genotype(s) of the parasite responsible for human hydatidosis in North India.

**Methodology/Principal Findings:**

To study the transmission patterns of *E. granulosus*, genotypic analysis was performed on hydatid cysts obtained from 32 cystic echinococcosis (CE) patients residing in 7 different states of North India. Mitochondrial *cytochrome c oxidase* subunit1 (*cox1*) sequencing was done for molecular identification of the isolates. Most of the CE patients (30/32) were found to be infected with hydatid cyst of either G3 (53.1%) or G1 (40.62%) genotype and one each of G5 (cattle strain) and G6 (camel strain) genotype.

**Conclusions/Significance:**

These findings demonstrate the zoonotic potential of G1 (sheep strain) and G3 (buffalo strain) genotypes of *E. granulosus* as these emerged as predominant genotypes infecting the humans in India. In addition to this, the present study reports the first human CE case infected with G5 genotype (cattle strain) in an Asian country and presence of G6 genotype (camel strain) in India. The results may have important implications in the planning of control strategies for human hydatidosis.

## Author Summary

Cystic echinococcosis caused by *Echinococcus granulosus* is a widespread zoonotic disease. This cestode parasite is a complex of genetic variants and to date, ten distinct genotypes (genotypes G1–G10) have been identified on the basis of mitochondrial DNA sequences. The strain variation in *E. granulosus* may influence life-cycle patterns, host range, antigenicity and transmission dynamics. The disease is endemic in different parts of India. Although the presence of G1, G2, G3 and G5 has been reported in animal isolates in India, the zoonotic potential of the different genotypes has not been evaluated. In the present study, a genotype analysis was carried out in hydatid cysts collected from 32 cystic echinococcosis patients residing in North India. Most of the CE patients (30/32) were found to be infected with hydatid cyst of either G3 (53.1%) or G1 (40.62%) genotype and one each of G5 (cattle strain) and G6 (camel strain) genotype.

## Introduction

Cystic echinococcosis (CE), caused by the metacestode of *Echinococcus granulosus*, is an important zoonosis that affects human and ungulate animals worldwide [1]. Although, the infection in domestic animals is usually asymptomatic and detected only at post-mortem inspection at the slaughter houses, yet it causes great economic loss through condemnation of infected organ, in particular liver [2]. *E. granulosus* requires dog and other canids as definitive hosts and livestock as intermediate host to complete its life cycle [3]. Human act as accidental intermediate host and become infected with food or water contaminated with feces of dog containing eggs of parasite or with direct contact with dogs [4]. Eggs hatch in small intestine and parasite larvae can reach to almost any organ, most commonly the liver, where they develop, form cysts and may remain asymptomatic for years. In symptomatic patients, infection may lead to symptoms of space occupying lesion due to cyst pressure on the surrounding tissues/organs or due to cyst rupture [5].

*E. granulosus* is a complex of species/strains which exhibit diversity in their life cycle patterns and host range [6]. To date, 10 genotypes of *E. granulosus* have been identified by molecular genetic analysis using mainly mtDNA sequences [7], [8], [9]. It has been suggested that *E. granulosus* genotypes should be split into 4 species: *E. granulosus* sensu stricto (genotypes G1–G3), *E. equinus* (G4), *E. ortleppi* (G5) and *E. canadensis* (G6–G10) [10], [11]. *E. felidis* (lion strain) isolated from South Africa has been identified as independent taxon [12]. Except G4 genotype all other strains have been found to infect the humans. Globally, most human cases of CE have been found to be infected with sheep strain (G1) of *E. granulosus* [3].

Earlier studies from Western India demonstrated the presence of 4 different genotypes of the *E. granulosus* (genotype G1, G2, G3 and G5) in food producing animals [13], [14], [15]. The G1 and G3 genotypes have been demonstrated to infect the livestock in North India [16]. Hospital based studies and case reports revealed that the disease is endemic in many parts of India [17], [18], [19], [20]. However, to date, no information is available regarding genotypes of *E. granulosus* infecting human in India. Genotyping of human CE is useful to assess the data on parasite transmission patterns for epidemiological purposes and the human susceptibility to a particular genotype of *E. granulosus*. Therefore, genotyping of hydatid cysts collected from human CE patients residing in North India was carried out by partially sequencing of mitochondrial *cytochrome c oxidase* subunit I gene (*cox1*) to ascertain the strain of parasite responsible for human hydatidosis.

## Materials and Methods

### Ethics statement

The study was approved by the Institutional Ethics Review Committees of the Post Graduate Institute of Medical Education and Research (PGIMER), Chandigarh, Sher-i- Kashmir Institute of Medical sciences (SKIMS), Srinagar, Kashmir and Indira Gandhi Medical College and Hospital (IGMC), Shimla, India.

All adult subjects and parents/guardians of the children gave their written informed consent for surgery. Institutional Ethical Review Committees exempted the study from informed consent as it was not applicable because in this study specimen/cyst obtain post surgically were used. Patient information was obtained from medical records following the surgery. The information of patient has been sufficiently anonymised so that they cannot be identified.

#### Patients and cyst collection

**Figure 1:**
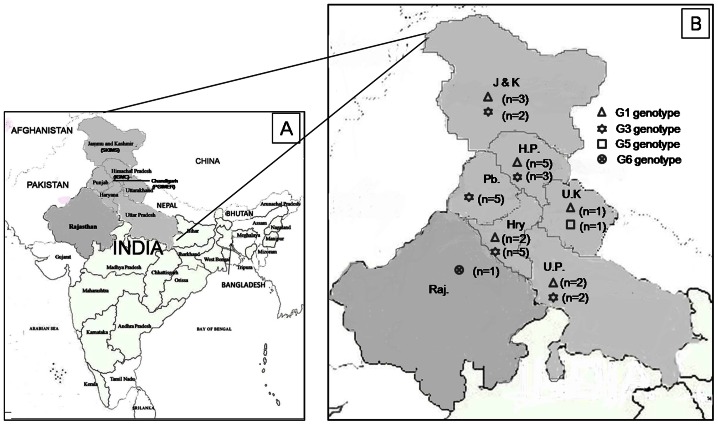
Geographical distribution of the cystic echinococcosis patients. A) Map of India showing the location of three hospitals and geographical origin of cystic echinococcosis patients (in grey) analyzed in this study. B) State wise geographical distribution of 4 different genotypes of *Echinococcus granulosus* in North India. The number of collected samples for each state is indicated. J& K indicates the Jammu and Kashmir, H.P - Himachal Pradesh, Pb - Punjab, Hry - Haryana, U.K - Uttarakhand, U.P - Uttar Pradesh, Raj-Rajasthan, PGIMER-Post Graduate Institute of Medical Education and Research, SKIMS - Sher-i- Kashmir Institute of Medical sciences, IGMC- Indira Gandhi Medical College and Hospital.

*E. granulosus* cysts were collected from 32 patients following surgical removal from three hospitals in North India (Fig. 1A). The cysts were collected during 2011–12 from clinically and radiologically suspected and/or seropositive hydatidosis patients who underwent surgery for hydatidosis. All the cysts removed surgically were confirmed morphologically by microscopical examination. The details of demographic and clinical data obtained from all the patients (age, sex, geographical area, cyst location, size of cyst) were recorded.

Nehru hospital attached to Post Graduate Institute of Medical Education and Research (PGIMER), Chandigarh is a tertiary care referral hospital and patients from different states in North India attend the hospital. Cysts were collected from 25 patients attending Nehru hospital, who were inhabitants of Punjab (n = 5), Haryana (n = 7), Himachal Pradesh (n = 5), Jammu and Kashmir (1), Uttarakhand (2), Uttar Pradesh (n = 4) and Rajasthan (n = 1) states. Travel history was obtained from these patients which indicated their stay within their hometown. Only one female patient was initially inhabitant of Allahabad and subsequently settled in Punjab state about one and half year, prior to attending PGIMER, Chandigarh for treatment.

Shimla, Himachal Pradesh and Srinagar, Jammu and Kashmir states are hilly sheep rearing areas in North India with high endemicity for human hydatidosis. Cysts were collected from 4 and 3 patients attending Sher-i- Kashmir Institute of Medical sciences (SKIMS), Srinagar, Kashmir and Indira Gandhi Medical College and Hospital (IGMC), Shimla, Himachal Pradesh respectively and transported to PGIMER, Chandigarh under refrigerated conditions for further processing. These patients were the local inhabitants. A total of 36 hydatid cysts samples (31 endocyst and 5 cyst fluid) were obtained from 32 hydatidosis patients after surgical resection for the treatment of CE.

All cyst samples were examined under microscope for the presence of protoscolex. Cyst fluids were centrifuged at 5000 g for 30 min. The pellet and germinal membrane were fixed in 95% ethanol and stored at −20°C till further use.

### Molecular analysis

For DNA extraction, cyst samples were washed thrice in PBS to remove ethanol and genomic DNA was extracted from each sample by QIAamp DNA mini kit (Qiagen, Hilden, Germany), according to the manufacturer's instructions.

For molecular identification, PCR amplification of the *cox1* gene was performed by using primers and PCR conditions as described previously [7] with minor changes. Briefly, amplification was performed in 50 µl final volume containing 2 µl DNA, 0.2 mM premixed solution of dNTP, 10 pmol of each primer, 1× PCR buffer, and 1 U of TaqDNA polymerase. Amplification program included an initial denaturation step of 95°C for 5 min and 38 cycles each of denaturation (95°C for 50 s), annealing (57°C for 50 s), extension (72°C for 1 min) and final extension of 72°C for 10 min. After agarose gel electrophoresis (1.5%), PCR products were purified and sequenced.

### Sequence analysis

**Figure 2:**
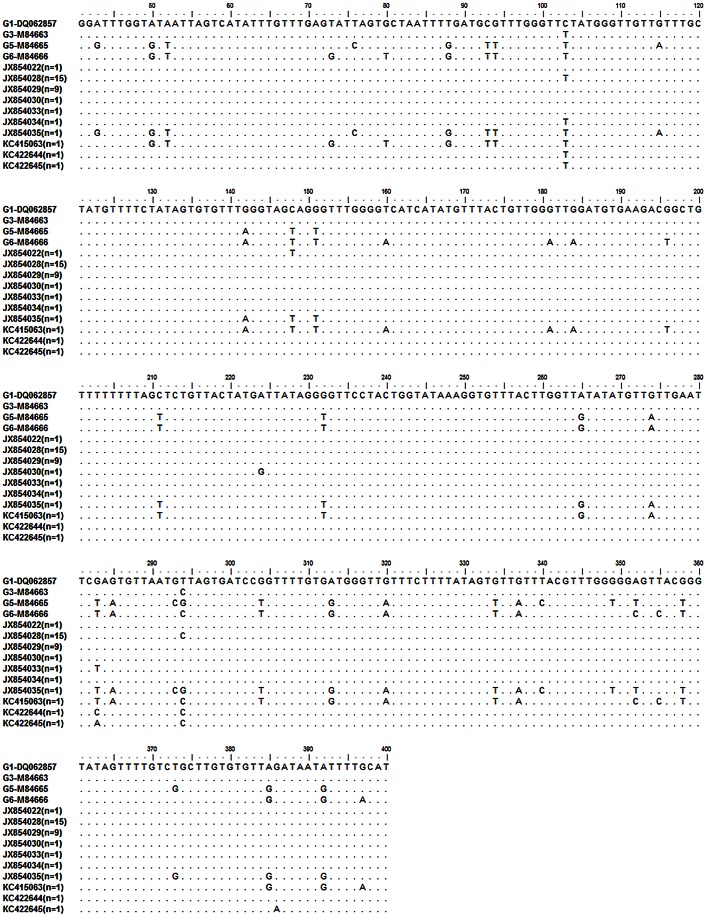
Multiple alignments of partial *cox1* gene sequences. Accession numbers with suffix JX and KC are the representative sequences of G1, G3, G5 and G6 genotypes and their haplotypes found in this study. Accession numbers DQ062857, M84663, M84665 and M84666 are the respective references sequences of G1, G3, G5 and G6 genotypes of *E. granulosus* retrieved from GenBank for comparison. n indicates the number of isolates detected for each haplotype.

Previously published sequences of *E. granulosus* isolates retrieved from the National Center for Biotechnology (http://www.ncbi.nlm.nih.gov) were used as the reference sequence (Fig. 2). Nucleotide sequence analysis was performed with BLAST sequence algorithms and sequences were aligned using ClustalW [21].

## Results

Hydatid cysts removed surgically from 32 cystic echinococcosis (CE) patients residing in 7 different states of North India were included in this study (Fig. 1A & 1B). The demographic characteristics of these CE patients are detailed in Table 1. Microscopical examination of all the cysts obtained post operatively, showed the presence of protoscolex and thus patients were confirmed of hydatidosis.

**Table 1:** Clinical characteristics of 32 cystic echinococcosis patients.

	Number of patients (%)	Genotype assigned
**Age (years)**		
10–20	9 (28.12)	G1,G3
>20–30	10 (3.12)	G1,G3,G5,G6
>30–40	7 (21.8)	G1,G3
>40–50	3 (9.37)	G1,G3
>50–70	3 (9.37)	G1,G3
**Sex**		
Male	19 (59.37)	G1, G3, G5, G6
Female	13 (40.62)	G1, G3
**Cyst Location**		
Liver	24 (75)	G1,G3, G5
Lung	4 (12.5)	G1, G3
Peritoneal cavity	1 (3.12)	G3
Brain	1 (3.12)	G6
Liver+Lung	1 (3.12)	G3
Liver+spleen	1 (3.12)	G1
**Cyst size**		
Large(>10 cm)	9 (28.12)	G1,G3
Medium(5–10 cm)	17 (53.12)	G1,G3
Small(<5 cm)	6 (18.75)	G1,G3,G6

The most common clinical manifestation in case of hepatic cyst was epigastric pain in 96.4% of patients; other complaints included abdominal distention, hepatomegaly, palpable abdominal mass, etc. In case of pulmonary cyst, patients presented with chest pain, cough and short breathlessness. Hemoptysis was observed in 2 patients. In case of intracerebral cyst, patient presented with severe headache associated with nausea, loss of appetite and vomiting.

### Molecular analysis

The amplification of *cox1* gene with JB3 and JB4.5 primers yielded amplification product of 446 bp. Nucleotide sequence of all the 32 isolates from North India were aligned with reference sequence of each genotype within *E. granulosus* retrieved from GenBank (Fig. 2). Sequence analysis revealed the presence of 4 genotypes of *E. granulosus*: sheep strain (G1 genotype), buffalo strain (G3 genotype), cattle strain (G5 genotype) and camel strain (G6 genotype).

Among 32 CE patients, 17 were found to be infected with cyst of G3 (53.1%) genotype, 13 with G1 (40.62%) genotype and the remaining 2 were found to be infected with G5 and G6 genotypes, one each. Comparison of sequence revealed that 9 and 15 isolates showed 100% homology with G1 (DQ062857) and G3 (M84663) genotypes respectively, while the sequence of other 6 isolates within G1 and G3 genotypes showed point mutation (Fig. 2). Specifically, one cyst sample (accession number JX854034) has thymine at position 294 like G1genotype, while rest of the sequence was identical to G3 genotype. Thus within G1 and G3 genotypes total 8 haplotypes were detected.

Only one sequence (accession number JX854035) showed 100% homology with the sequence selected as reference for the G5 genotype (cattle strain). The cyst of this genotype was from a 21 year old man who underwent surgery for hepatic hydatid cyst (6×4 cm in size) located in segment V and reaching into segment VI of liver. The sequence of another isolate (accession number KC415063) showed 100% homology to reference sequence of G6 genotype. The cyst of this genotype was isolated from the brain of 22 year old man. In 4 patients more than one cyst were isolated and all the cysts from the same individual, belonged to same genotype, showing 100% homology among themselves.

### Genotypes and their geographical location

All the patients from Punjab state (5/5) were found to be infected with hydatid cyst of G3 genotype. Patients from Haryana, Himachal Pradesh, Jammu and Kashmir and Uttar Pradesh states were found to be infected with hydatid cyst of either G1 or G3 genotypes, whereas the patients from Uttarakhand harbored the cyst of either G1 or G5 genotype. One patient from Rajasthan was infected with cyst of G6 genotype (Fig. 1B).

## Discussion

Hydatid disease is endemic in many parts of India and is a cause of serious concern due to increase in morbidity and considerable economic loss. In India several factors including cultural, educational, socioeconomical, agricultural and environmental contribute to the transmission of infection [22]. In addition to this, lack of education and knowledge about the life cycle of the *E. granulosus*, as well as the lack of legislation for meat inspection and offal disposal at local abattoirs, contributes to domestic cycles of transmission [23]. Genotyping of human cases of CE play an important role in the formulation of control strategies for the prevention of transmission of this parasite. Strain variation in parasites exhibit variation in the onset of egg production, which is a limiting factor in control programs, which employs adult cysticidial treatment of definitive host to break the cycle of transmission [24]. Further it is postulated that the strain variation in parasite may influence host specificity, life-cycle patterns, development rate, transmission dynamics, antigenicity and sensitivity to chemotherapeutic agents. Therefore it may have implications for the development and design of vaccines and diagnostic reagents [4].

Pednekar et al. [15] reported four genotypes of *E. granulosus* namely the sheep strain (G1), Tasmanian sheep strain (G2), Indian buffalo strain (G3) and cattle strain (G5) of *E. granulosus* in livestock in Maharashtra and adjoining areas in Western India. The predominant genotype was found to be G3 genotype (63%) present in all species of livestock followed by the G5 (19.56%), the G1 (13%) and the G2 genotype (4.34%). In Ludhiana, North India, buffalo strain (G3) and common sheep strain (G1) was found to infect the livestock [16]. Information about the genotypes of *E. granulosus* infecting the human population are lacking in India. Therefore to find out the possible relevance of these strains in human cystic echinococcosis cases, genotyping analysis was performed on 32 North Indian patients, who underwent surgery for the removal of hydatid cyst.

In concordant to the earlier studies from livestock (cattle, buffalo, pig and sheep) in India [15], [16], in the present study, the buffalo (G3 genotype) and sheep strains (G1 genotype) were found as predominant genotypes in human cystic hydatid cases. In India both G1 and G3 genotypes were found to be commonly encountered and are well adapted to different ungulates animals (cattle, buffalo, sheep, goats and pigs) [15]. The sheep strain (G1 genotype) has always been considered as the major source of human infection worldwide [25], [26], [27]. The buffalo (G3) strain has been demonstrated to infect the human in Europe and South America [28], [29]. The G3 genotype was also found in different intermediate hosts in European countries [28], [30].

In contrast to an earlier study from Western India [15], reporting that after the G3 genotype, G5 genotype was found as second most common genotype in livestock (cattle, buffalo and pig), while in the present study the G5 genotype (cattle strain) was detected in liver of only one of human isolate. This suggests a low pathogenic risk of cattle strain for human as compared to sheep and buffalo strain. In past years, only a few cases of G5 genotype have been identified in humans: one patient from Netherland harboring cyst in spleen and another from Mexico had cyst in right lobe of the liver [31], [32]. Molecular investigations of cystic echinococcosis patients from Argentina and Southern Brazil also revealed the presence of this genotype only in 1/66 and 1/6 cases respectively [29], [33]. However, the likely location of cysts in these patients was not described. The G5 genotype was also found to infect the livestock in South America, Iran and India [15], [33], [34].

The G6 (camel strain) genotype was found in one human isolate in the present study. The patient is residing in Rajasthan state where camels are frequently found, thereby suggesting camel as an alternative host for the transmission of human hydatidosis in this region. This genotype was also found to infect humans in Eastern Africa, Brazil, South-eastern Romania, Turkey and China [35], [36], [37], [38], [39]. The presence of G6 genotype in goats in Argentina and Kenya indicates goat as reservoir of camel strain [40], [41]. The present study also revealed the first evidence of G6 genotype in India.

Comparison of geographical data with molecular analysis in the present study have shown that all the CE patients from Punjab had the cyst of G3 genotype, whereas patients from Haryana, Himachal Pradesh, Uttar Pradesh, Uttarakhand exhibited the cyst of either G1 or G3 genotype. G5 genotype was found in only one patient from Uttarakhand. Thus both G1 and G3 genotypes showed a wide geographical distribution in North India, being distributed in 5 (Haryana, Himachal Pradesh, Srinagar, Uttar Pradesh and Uttarakhand) and 4 states (Punjab, Haryana, Himachal Pradesh, Srinagar, Uttar Pradesh) respectively. In 4 patients, more than one cyst were isolated and all the cysts from the same individual, belonged to same genotype, showing 100% homology in nucleotide sequence among themselves, which indicates a single source of infection from definitive host. This finding is consistent with the study reported from China, where multiple cysts were obtained from 13 patients and the same individuals were harboring the cysts of same genotype [35]. As reviewed by Traub et al. [42] the prevalence of CE infection in dogs varies from 3.5% to 33%. Pednekar et al. [15] also reviewed various studies carried out between 1980–2008 which showed a high prevalence of this disease in livestock in India: 2.5–50% in sheep, 4–48.1% in buffaloes, 1.7%–45 in cattle, 0–11.25% in pigs. Altogether, these data demonstrate the wide dissemination of *E. granulosus* between dogs as definitive host and other intermediate hosts (sheep, buffalo, cattle and pigs) in India thus increasing the hazardous of infection to humans. As the majority of CE patients (30/32) harbored the cyst of either G1 or G3 genotype, therefore no specific correlations between patient's demographic characteristics, clinical data with the genotypes of corresponding hydatid cysts (G1, G3, G5 and G6) could be evaluated.

To the best of our knowledge, this study is the first report of genotyping of hydatid cyst of *E. granulosus* from cystic hydatidosis patients in India. In conclusion, the result of the present study demonstrated the zoonotic potential of G1 (sheep strain) and G3 (buffalo strain) genotypes as these emerged as predominant genotype infecting the humans. The study illustrates the first human cystic hydatidosis case of G5 genotype (cattle strain) in Asian countries and the presence of G6 genotype (camel strain) in India. The results may have important implications in the planning of control strategies for human hydatidosis.

### Accession numbers of *cox 1* gene of different genotypes of *Echinococcus granulosus*

Accession numbers of reference sequence of *E. granulosus*: G1 genotype - DQ062857, G3 genotype - M84663, G5 genotype - M84665, G6 genotype - M84666.

Accession numbers of isolates analysed in this study: Haplotypes of G1 and G3 genotypes: JX854022, JX854028, JX854029, JX854030, JX854033, JX854034, KC422644, KC422645 G5 genotype -JX854035, G6 genotype -KC415063
